# Association of intratumoral microbiome diversity with hepatocellular carcinoma prognosis

**DOI:** 10.1128/msystems.00765-24

**Published:** 2024-12-11

**Authors:** Fengle Jiang, Yuan Dang, Zheting Zhang, Yanan Yan, Yingchao Wang, Yi Chen, Lihong Chen, Jialiang Zhang, Jingfeng Liu, Jianmin Wang

**Affiliations:** 1 Innovation Center for Cancer Research, Clinical Oncology School of Fujian Medical University, Fujian Cancer Hospital, Fuzhou, China; 2 Fujian Key Laboratory of Advanced Technology for Cancer Screening and Early Diagnosis, Fuzhou, China; 3 The United Innovation of Mengchao Hepatobiliary Technology Key Laboratory of Fujian Province, Mengchao Hepatobiliary Hospital of Fujian Medical University, Fuzhou, China; 4 Department of Hepatopancreatobiliary Surgery, Clinical Oncology School of Fujian Medical University, Fujian Cancer Hospital, Fuzhou, China; E O Lawrence Berkeley National Laboratory, Berkeley, California, USA

**Keywords:** intratumoral microbiome, hepatocellular carcinoma, 16S rRNA gene sequencing, bacterial components, tumor-associated immune cells

## Abstract

The evidence that intratumoral microbiomes, as a rising hallmark of cancer, have a profound impact on cancer phenotypes is increasingly compelling. However, the impact of the composition and diversity of the intratumoral microbiome on the prognosis of patients undergoing surgical resection for hepatocellular carcinoma (HCC) remains incompletely understood. In this study, we revealed a high abundance of bacteria in the neoplastic tissues. The presence of bacterial lipopolysaccharide and lipoteichoic acid was detected alongside tumor-associated immune cells. By utilizing 16S rRNA gene sequencing, we identified a specific intratumoral microbiome signature that was highly predictive of the prognosis for HCC patients who underwent surgical resection. Specifically, the presence of Intestinimonas, Brachybacterium, and Rothia were identified as independent risk factors for the overall survival of HCC patients who underwent surgical resection.

**IMPORTANCE:**

Although some studies have shown an abundance of bacteria in hepatocellular carcinoma (HCC), there is still limited understanding of the composition and diversity of the intratumoral microbiome that is favorable or adverse to the prognosis of HCC patients. Our results indicated that a greater abundance of bacteria could be observed in the neoplastic tissues than in nonneoplastic tissues. Bacterial cell wall components largely coincided with tumor-associated immune cells. The bacteria in the long overall survival (LOS) group were associated with metabolism and cytokine‒cytokine receptor interaction pathways, while bacteria in the short overall survival (SOS) group were associated with proinflammatory and cell proliferation pathways. Notably, specific taxa could independently predict HCC prognosis. Based on these findings, intratumoral microbiomes facilitate the use of precision medicine in clinical practice.

## INTRODUCTION

Primary liver cancer is currently the fourth most common malignant tumor and the second leading cause of tumor-related deaths in China (1–3). It primarily includes hepatocellular carcinoma (HCC), intrahepatic cholangiocarcinoma (ICC), and mixed hepatocellular carcinoma-cholangiocarcinoma (cHCC-CCA). These three types vary greatly in their pathogenesis, biological phenotype, pathological histology, treatment methods, and prognosis. Among them, HCC accounts for 75% to 85% of primary liver cancers (4). HCC treatment is multidisciplinary and involves multiple treatments, including hepatectomy, radiofrequency or microwave ablation, liver transplantation, transarterial chemoembolization (TACE), transarterial radioembolization (TARE), and stereotactic body radiation (SBRT) (5). In recent years, immunotherapy has shown persistent efficacy in HCC treatment (6). Surgical treatment, including hepatectomy and liver transplantation, provides the best opportunity for achieving long-term survival in HCC patients. The overall survival rates for HCC patients who underwent hepatectomy are 73.2%, 28.8%, and 19.6% at 1, 3, and 5 years, respectively (7). However, current clinical features and biomarkers, such as the TNM staging system, Barcelona Clinic Liver Cancer (BCLC) staging system, and serum alpha-fetoprotein (AFP) level, are insufficient for providing accurate prognostic evaluations for HCC patients in clinical practice (8, 9). For example, the serum AFP level, which is the most widely used marker, has been found to be nearly half-negative in HCC patients with early-detected and small tumors (10). Moreover, the TNM and BCLC staging systems are not universally applicable to HCC patients due to their various etiologies and genetic backgrounds (8).

The evidence that intratumoral microbiomes, as a rising hallmark of cancer, have a profound impact on cancer phenotypes is increasingly compelling (11–14). Recent research has revealed that various bacteria are abundant in various tumor tissues (15). However, the tumor microbiome remains incompletely characterized due to technological limitations in analysis. Nejman et al. demonstrated close associations between different tumor types, their microbiomes, bacterial metabolism, and clinical features. They also identified cancer-type-specific microbial signatures across seven types of human tumors (15). Chai et al. investigated the intratumoral microbiota profile and demonstrated the antitumor effect of *Paraburkholderia fungorum* in ICC (16). Huang et al. developed a machine-learning model for predicting HCC prognosis based on intratumoral bacterial features, identifying specific taxa as potential therapeutic and diagnostic targets (17). Chen et al. revealed correlations among microbial species, metabolites, DNA methylation, and gene expression alterations, suggesting the potential of intratumoral bacteria as novel biomarkers and therapeutic targets for HCC (18, 19). Additionally, the microbiota has been found to be associated with the tumor microenvironment, with bacteria acting as antigens that influence the immune response (20). The intratumoral microbiota can reshape the tumor microenvironment, thereby impacting cancer progression (21). Notably, Toll-like receptor 2 senses bacterial lipopolysaccharide (LPS) and greatly contributes to the generation of an immunosuppressive microenvironment, ultimately promoting cancer progression (22, 23). Moreover, bacteria metabolize chemotherapeutic drugs, affecting the efficacy of cancer therapies (24).

The current understanding of the effect of the intratumoral microbiome on HCC prognosis remains limited. In this study, we aimed to address this gap by analyzing the microbial spectrum of human HCC based on 16S rRNA gene sequencing of 172 tumor tissues and their adjacent normal tissues. Our findings revealed that intratumoral bacteria were predominantly present in cancer and tumor-associated immune cells. Moreover, we identified specific microbial signatures that could serve as prognostic indicators for HCC in clinical practice.

## RESULTS

### Composition of the intratumoral microbiota in HCC tissues

**Fig 1:**
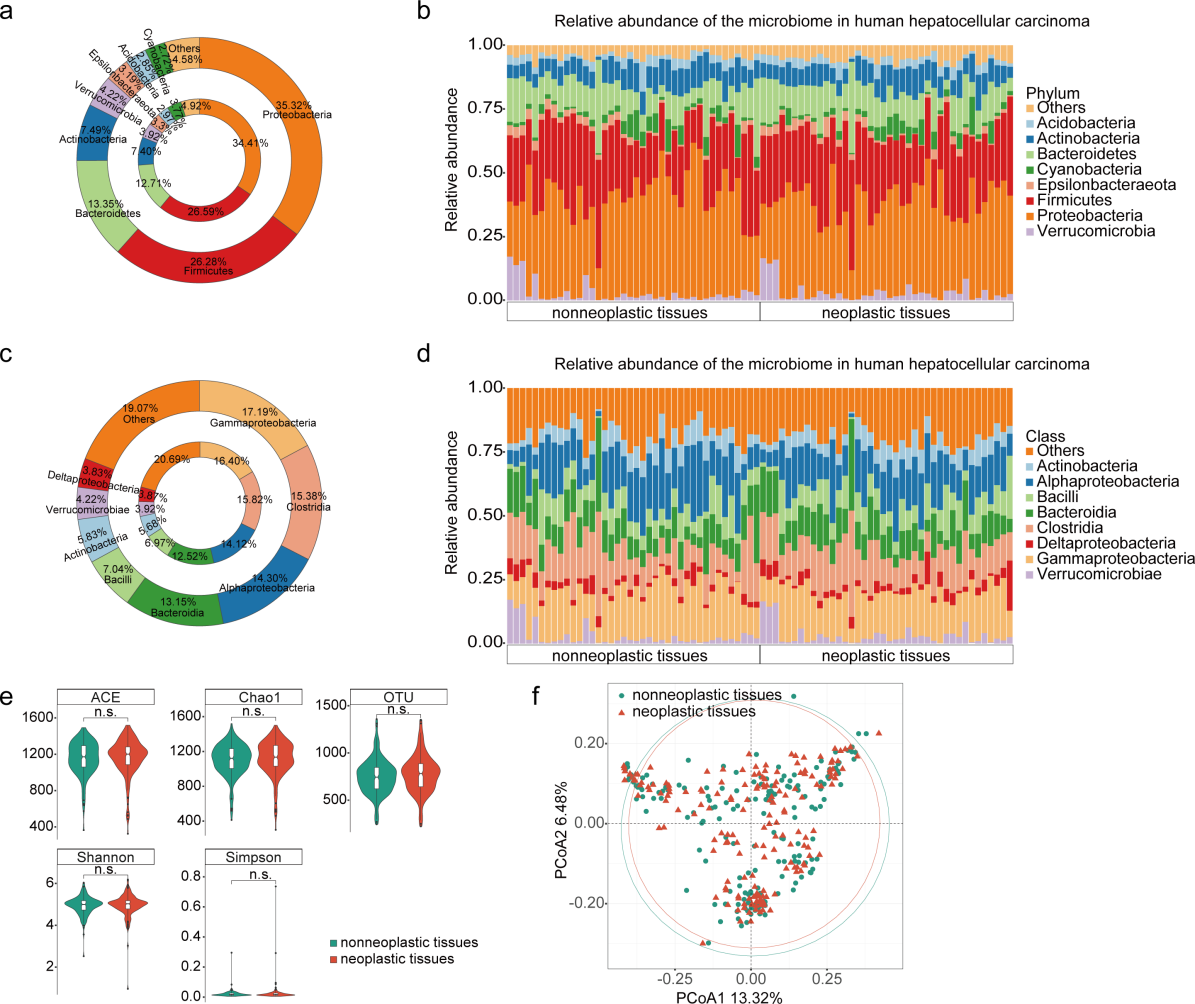
Characterization of bacteria in HCC tissues. (**a and **b) Pie plots of bacteria at phylum level in neoplastic tissues and nonneoplastic tissues. (**c and** d) Pie plots of bacteria at the class level in neoplastic tissues and nonneoplastic tissues. (e) Alpha diversity box plot (ACE, Chao1, OTU, Shannon, and Simpson indices) of neoplastic tissues and nonneoplastic tissues. (f) Beta diversity of neoplastic tissues and nonneoplastic tissues.

To explore the composition of the intratumoral microbiota in surgically resected HCC tissues, we enrolled 172 patients with HCC from Mengchao Hepatobiliary Hospital of Fujian Medical University in Fujian Province, China. Bacterial RNA was extracted from surgically resected HCC tumors and adjacent normal tissues, and taxonomic profiling via 16S rRNA gene sequencing was performed. A total of 23,703,176 sequencing reads with an average length of 417 bp were obtained. These reads corresponded to 1,752 operational taxonomic units (OTUs) at various taxonomic levels, including 109 species, 486 genera, 233 families, 139 orders, 63 classes, 28 phyla, and 2 kingdoms (Table S1A through C). Proteobacteria, Firmicutes, Bacteroidetes, Actinobacteria, and Verrucomicrobia were the predominant phyla observed (Fig. 1a and b), while Gammaproteobacteria, Clostridia, Alphaproteobacteria, Bacteroidia, Bacilli, Actinobacteria, Verrucomicrobiae, and Deltaproteobacteria were the most abundant classes (Fig. 1c and d). Our results revealed a microbial composition that was largely similar between HCC and adjacent normal tissues. Subsequently, we assessed tumor microbial diversity using different methodologies. The alpha diversity indices (ACE, Chao1, OTU, Shannon, and Simpson indices), indicating the number of species present within each tumor sample, were comparable between the neoplastic tissues and nonneoplastic tissues (*P* = 0.305, *P* = 0.160, *P* = 0.140, *P* = 0.558, and *P* = 0.803 for each alpha diversity index, respectively) (Fig. 1e). Additionally, we evaluated the beta diversity of community species by using principal coordinates analysis (PCoA), which characterizes similarities or differences in community composition among different subgroups. As shown in Fig. 1f, the intratumoral microbial composition of neoplastic tissues closely resembled that of adjacent nonneoplastic tissues (*P* > 0.05), suggesting phylogenetic closeness within each group.

### Presence of bacterial RNA in HCC tumors

**Fig 2:**
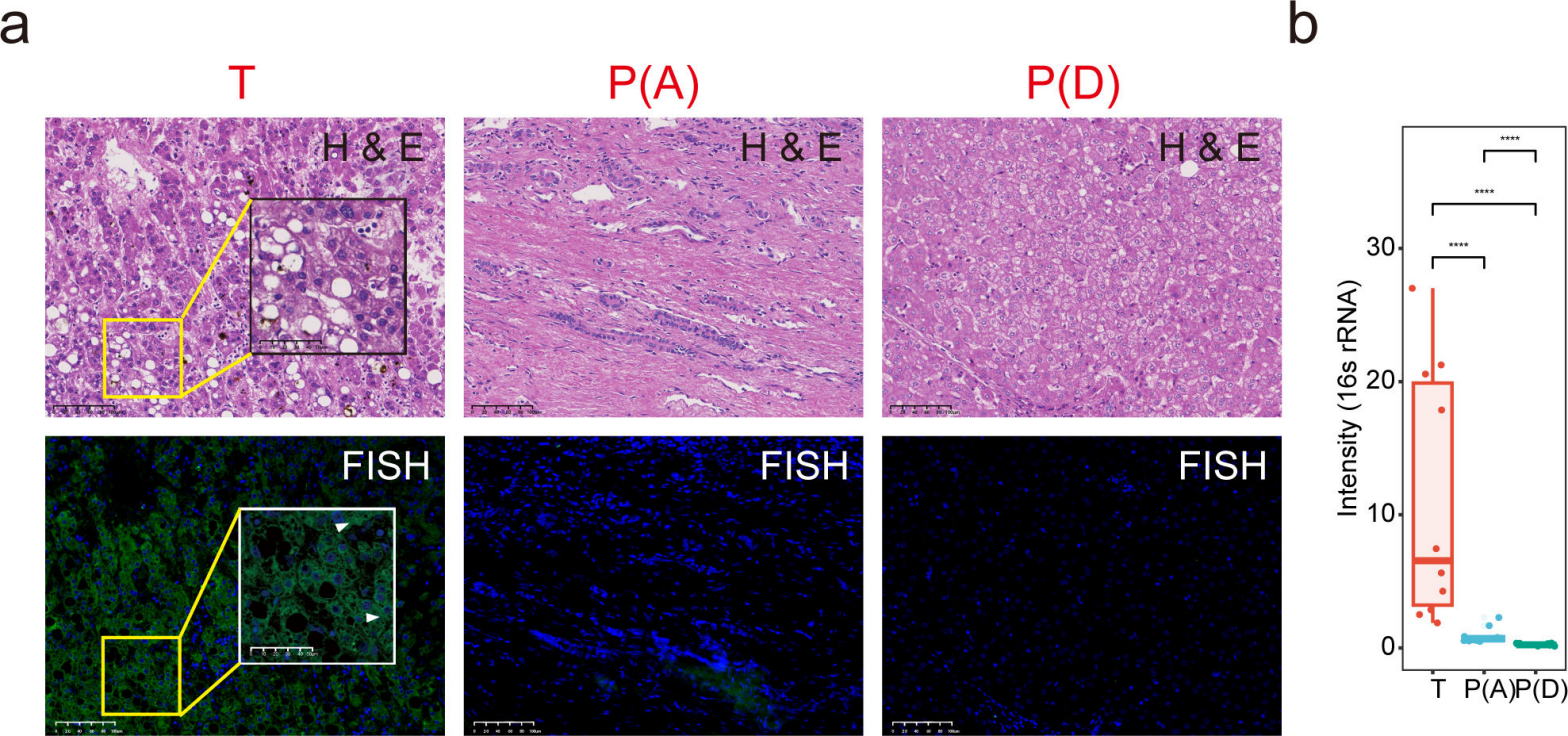
Bacterial RNA is present in HCC tissues. (a) Consecutive slices from the HCC sample were stained with H&E and FISH probes against bacterial RNA; imaging at 20×. Bacterial 16S rRNA was stained in green, and cellular nuclei were stained in blue. The yellow box indicates the region enlarged in the low right. The white arrows indicated the 16S rRNA signal in the slices. Scale bars, 50 µm. (b) The boxplot of bacterial RNA intensity. Thirty regions of interest (ROIs) were selected. T, tumor tissue; P(A), nonneoplastic tissue (adjacent); P(D), nonneoplastic tissue (distant).

To detect the presence of bacterial RNA in tumor tissues, consecutive slices of HCC tissues were stained with hematoxylin and eosin (H&E), and fluorescence *in situ* hybridization (FISH) was performed with a universal probe against bacterial 16S rRNA. In our study, bacterial RNA was frequently detected in neoplastic tissues but was only occasionally detected in adjacent nonneoplastic tissues. However, bacterial RNA was seldom detected in the region of the distant nonneoplastic tissues (Fig. 2a and b; Fig. S1 and Table S2).

### Presence of bacterial lipopolysaccharide and lipoteichoic acid inside HCC tumors

**Fig 3:**
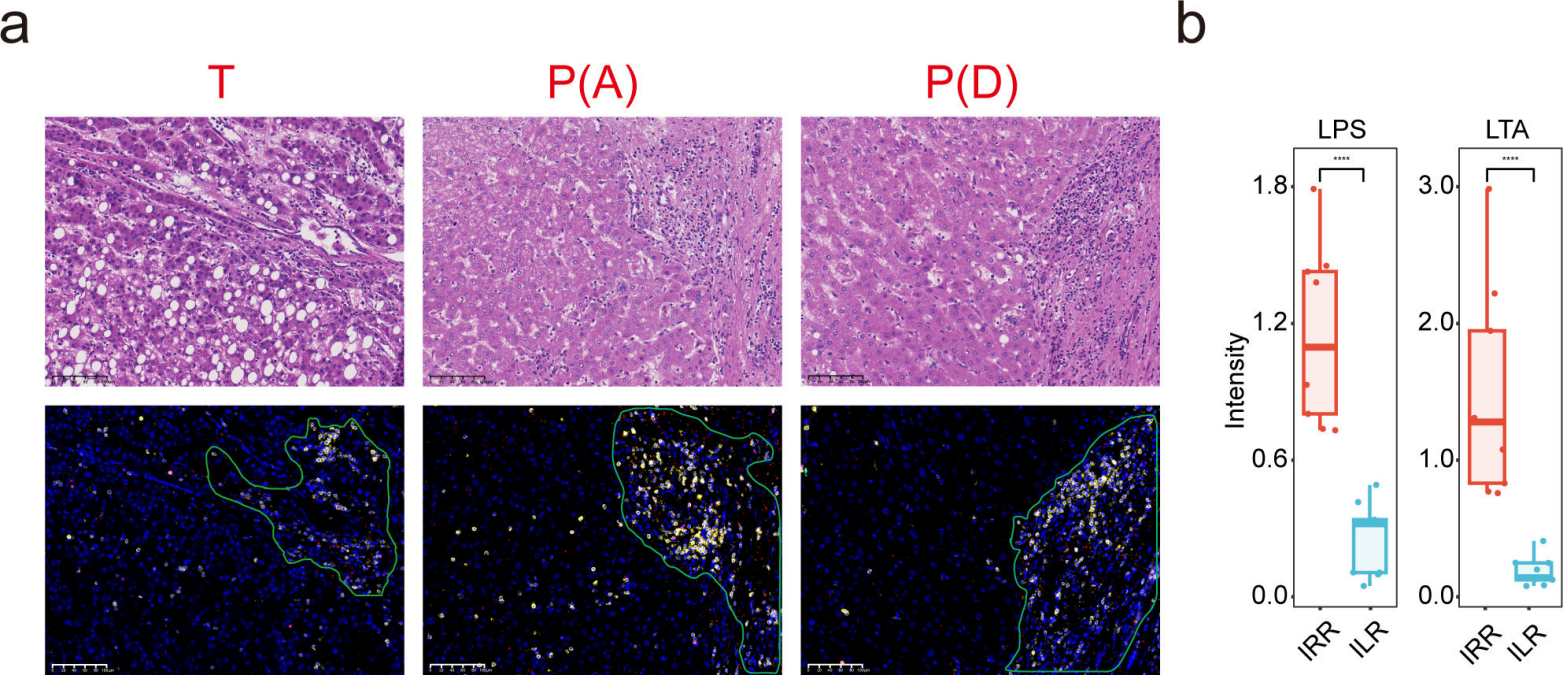
Bacterial LPS and LTA are present inside HCC tissues. (a) Representative images of multiplex immunofluorescence (multiplex IF, 20×) staining with an Opal kit. The bacteria were stained with antibodies against LPS and LTA, while CD45 and CD68 were used to identify immune cells. The area circled by the green curve indicates the IRR. Scale bars, 50 µm. (b) The boxplot of bacterial LPS and LTA intensity. Nine ROIs were selected.

To further validate the presence of bacteria in HCC tumors, bacterial LPS and lipoteichoic acid (LTA), which are from Gram-negative and Gram-positive bacteria, respectively, were detected using multiplex immunofluorescence. Unlike bacterial RNA, which was predominantly detected in the neoplastic tissues, bacterial LPS and LTA were observed in both the neoplastic and the nonneoplastic tissues (Fig. 3a and Fig. S2). As detected by H&E and IF for CD45, bacterial LPS, and LTA were mainly detected in immune cell-rich regions (IRR) compared to immune cell-lacking regions (ILR) (Fig. 3b; Table S3A and B). These results, along with FISH staining by 16S rRNA, confirmed the presence of bacteria inside the HCC tumor tissues.

### Diversity and similarity of intratumoral microbiome communities between the LOS and SOS patients

To explore the role of the human tumor microbiome composition in mediating the prognosis of HCC patients who underwent surgical resection, 172 patients were categorized into a long overall survival (LOS) group and a short overall survival (SOS) group based on 36 months survivorship after surgery. Patients in the LOS and SOS groups were matched for sex, age, tumor diameter, vessel invasion status, hepatitis B virus (HBV) infection status, TACE, and Huaier granule treatment (Table 1).

**TABLE 1:** Clinicopathological characteristics of HCC patients

Patient characteristic	SOS (*n* = 85)	LOS (*n* = 87)	*P* value
Overall survival (months)			<0.0001
Median	16.467	59.867	
Range	1.633–35.867	36.1–95.133	
Gender			0.263
Female	12	19	
Male	73	68	
Age (years)			0.165
Median	55	57	
Range	23–75	23–79	
Tumor diameter			2.118 × 10^−6^
Median	8	4	
Range	1.5–19	1.5–18	
Vessel invasion			1.127 × 10^−6^
Portal vein || hepatic vein	33	7	
Micrangium	39	45	
None	13	35	
HBV infection			0.0569
Yes	58	46	
No	27	41	
TACE			0.934
Yes	65	68	
No	20	19	
Huaier granules			0.551
Yes	29	25	
No	56	62	
Accompanying liver disease			0.197
Cirrhosis	78	73	
Fatty liver	3	9	
None	4	5	

**Fig 4:**
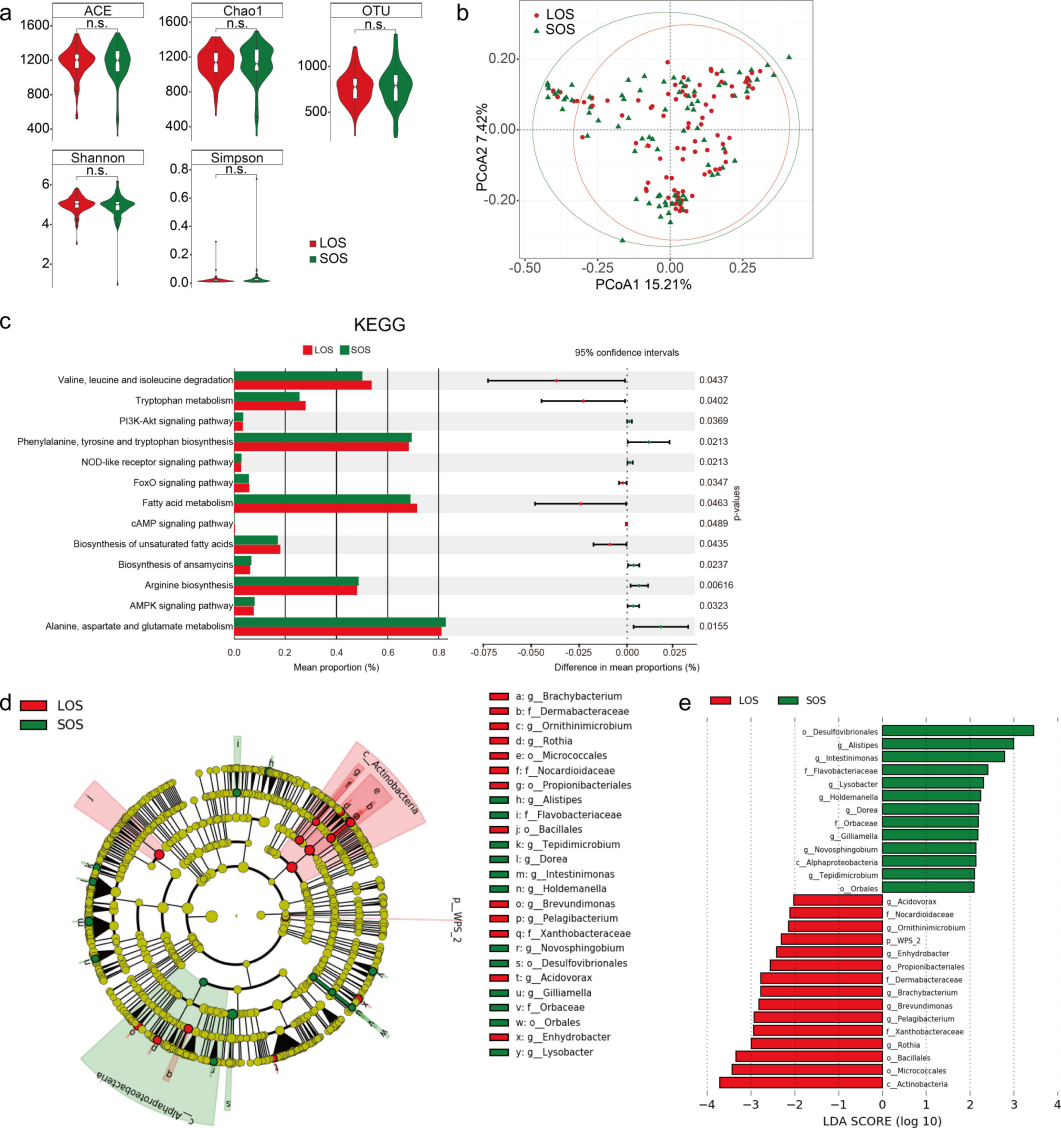
Diversity and similarity of intratumoral microbiome communities between the LOS and SOS patients. (a) Alpha diversity boxplot (ACE, Chao1, OTU, Shannon, and Simpson indices) of the LOS and SOS groups. (b) Beta diversity of the LOS and SOS groups. (c) PICRUSt2 combined with the Kyoto Encyclopedia of Genes and Genomes (KEGG) database to predict the function of the bacterial microbiota in LOS and SOS, showing the results of the level 3 KEGG pathway analysis. (d) Taxonomic cladogram from LEfSe, depicting the taxonomic association between microbiome communities from LOS and SOS HCC patients. Each node represents a specific taxonomic type. The yellow nodes denote the taxonomic features that were not significantly different between the LOS and SOS groups. Red nodes denote the taxonomic types with greater abundance in the LOS than in the SOS group, while green nodes represent the taxonomic types with greater abundance in the SOS group. (e) Linear discriminant analysis (LDA) score computed from features differentially abundant between the LOS and SOS groups. The criteria for feature selection were an LDA score > 2 and a Wilcoxon rank sum test *P* < 0.05.

Tumor microbial diversity between the LOS and SOS groups was assessed. We found that alpha diversity was similar among the LOS and SOS groups (*P* = 0.825, *P* = 0.626, *P* = 0.580, *P* = 0.273, and *P* = 0.270 for each alpha diversity index) (Fig. 4a). To further understand the role of microbiome diversity and its association with survival, we investigated whether phylogenetic relationships existed between the bacterial communities enriched in different tissues by using PCoA. As indicated, no significant difference in beta diversity between the LOS and SOS groups was observed in this cohort (*P* > 0.05, Fig. 4b).

ggPiCRUSt2 (25) was used to infer the function of the microbiota based on the OTU table. The predicted biological functions of bacteria were obtained by 16S amplicon sequencing with the Kyoto Encyclopedia of Genes and Genomes (KEGG, https://www.genome.jp/kegg/) (26) (Fig. 4c and Table S4). The analysis revealed that the FoxO signaling, fatty acid metabolism, and cAMP signaling pathways were enriched in the LOS group, while the PI3K-Akt signaling, NOD-like receptor signaling, and AMPK signaling pathways were enriched in the SOS group. This result indicated that the bacterial microbiota of the LOS group promoted fatty acid degradation, whereas the bacterial microbiota of the SOS group promoted cell proliferation and proinflammatory effects.

To further investigate these findings, high-dimensional class comparisons were conducted using LEfSe and the Wilcoxon rank sum test (LDA > 2, *P* < 0.05), which could reveal marked differences in the predominance of taxa between the LOS and SOS groups. The LOS group tumors exhibited a predominance of 15 categories, such as WPS_2 at the phylum level, Actinobacteria at the class level, and Rothia at the genus level. In contrast, the SOS group was dominated by 13 categories, such as Alphaproteobacteria at the class level and Intestinimonas at the genus level (Fig. 4d and e; Table S5).

### Intratumoral bacteria predict the prognosis of HCC patients

**Fig 5:**
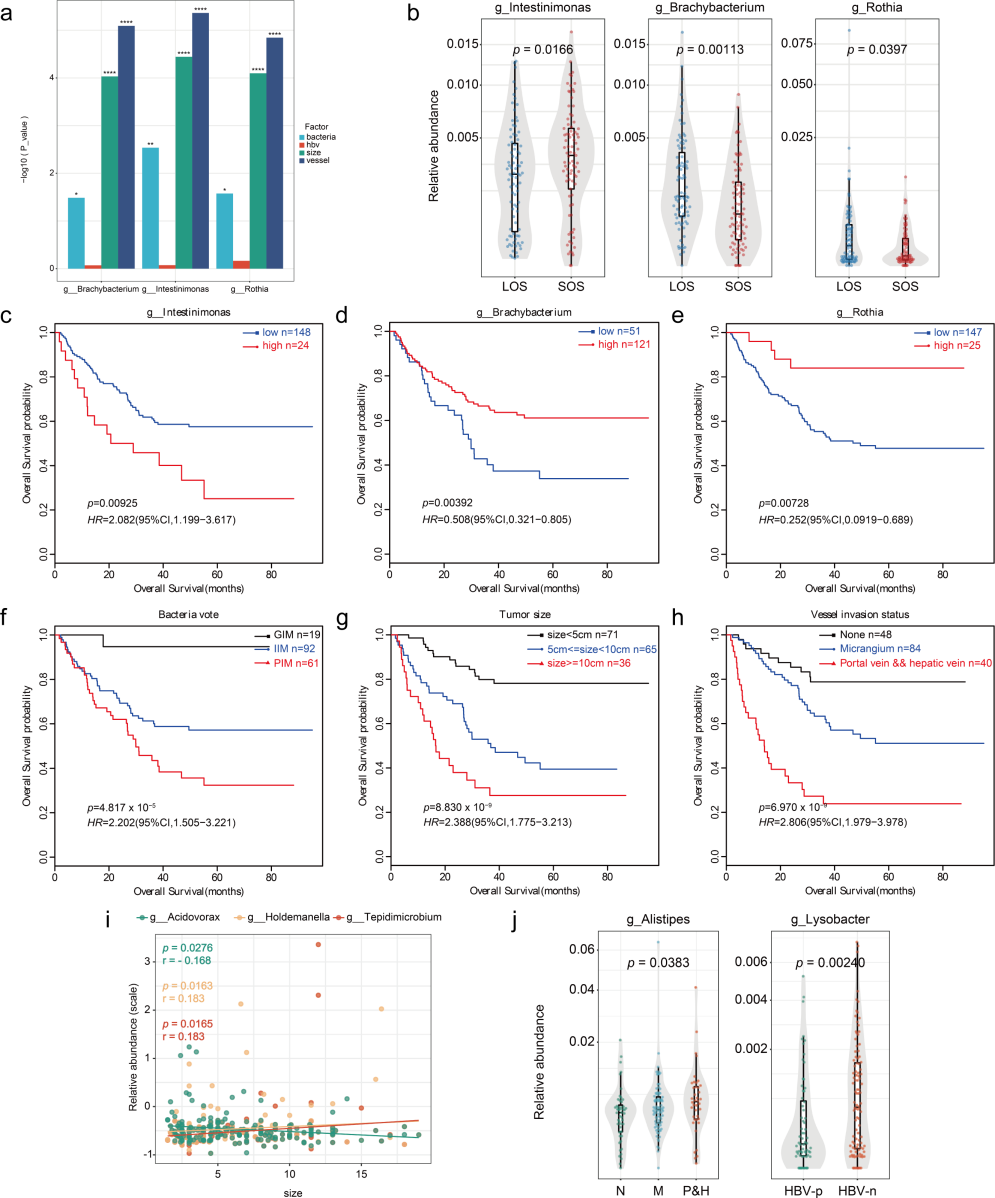
Intratumoral bacteria predicted HCC prognosis. (a) Multivariate Cox regression analyses for the abundance of bacteria, HBV status, vessel invasion status, and tumor size in the data set (*P < 0.05; **P < 0.01; ***P < 0.001). (b) Plots of the differentially abundant genera. *P* values were obtained from the Wilcoxon rank sum test. (**c–**e) Kaplan‒Meier estimates for survival probability based on the abundance of microbes enriched at the genus level. (c) Intestinimonas; (d) Brachybacterium; (e) Rothia (*P* < 0.005). (**f–**h) Kaplan‒Meier estimates for survival probability based on the abundance of the three microbes above, vessel invasion status, and tumor size. (f) Bacteria vote; (g) tumor size; (h) vessel invasion status (*P* < 0.0001). (**i and j**) Correlation analysis between key bacteria and clinical factors. (i) Tumor size; (j) vessel invasion status and HBV DNA status. N, none; M, micrangium; P&N, portal vein and hepatic vein; HBV-p, HBV DNA > 500; HBV-n, HBV-DNA < 500.

Among all the bacteria with significantly different abundance between the LOS and SOS groups, 15 categories were characterized at the genus level (Fig. 4d and e; Table S5). We conducted a multivariate Cox regression analysis on prognosis by using the 15 genera, together with HBV infection status and other clinicopathological features, to identify independent risk factors. Three taxa, namely, Intestinimonas, Brachybacterium, and Rothia, were found to be independent risk factors for overall survival (Fig. 5a). Intestinimonas was significantly more abundant in the SOS group compared to the LOS group, while Brachybacterium and Rothia were significantly less abundant in the SOS group than in the LOS group (Fig. 5b). The distributions of these three taxa significantly differed between the SOS and LOS groups.

We then stratified patients into high-risk versus low-risk groups based on these three taxa. A worse prognosis was predicted for HCC patients who underwent surgical resection and had a greater abundance of Intestinimonas (hazard ratio [HR] = 2.082, 95% confidence interval [CI] 1.199–3.617, *P* = 0.00925) and a lower abundance of Brachybacterium (HR = 0.508, 95% CI 0.321–0.805, *P* = 0.00392) and Rothia (HR = 1.518, 95% CI 0.960–2.401, *P* = 0.0740) (Fig. 5c thorugh e).

To explore the associations among these three taxa and the 5-year mortality of patients who underwent surgical resection, we utilized Fisher’s exact test to distinguish between the high-risk and low-risk groups defined above. A higher mortality rate was predicted for HCC patients who underwent surgical resection and had a greater abundance of Intestinimonas (odd ratio [OR] = 2.543, 95% CI 0.948–Inf, *P* = 0.0623). In contrast, lower mortality was predicted for HCC patients who underwent surgical resection and had a greater abundance of Brachybacterium (OR = 0.340, 95% CI 0–0.723, *P* = 0.00666) and Rothia (OR = 0.192, 95% CI 0–0.581, *P* = 0.00384) (Table S6).

To further characterize the potential for Intestinimonas, Brachybacterium, and Rothia as prognostic biomarkers, we classified patients into groups according to the presence of these three bacteria. The patients were classified as “poor prognosis predicted by intratumoral microbiome” if all three candidate taxa indicated that the patient was at high risk, while they were classified as “good prognosis predicted by intratumoral microbiome” if no candidate bacteria indicated that the patient was at high risk. The remaining patients were classified as “intermediate prognosis predicted by the intratumoral microbiome.” We found that the three taxa possessed discriminatory abilities for overall survival (HR = 2.202, 95% CI 1.505–3.221, *P* = 4.817 × 10^−5^), similar to the pattern for tumor diameter (HR = 2.338, 95% CI 1.775–3.213, *P* = 8.830 × 10^−9^) and vessel invasion status (HR = 2.806, 95% CI 1.979–3.978, *P* = 6.970 × 10^−9^) in predicting HCC prognosis (Fig. 5f through h). These results indicated that the combination of these three bacteria could contribute to prognosis determination.

Next, we investigated whether the abundance of the other 12 genera was associated with the clinicopathological parameters of HCC patients. We found that the abundances of Acidovorax (r = −0.168, *P* = 0.0276), Holdemanella (r = 0.183, *P* = 0.0163), and Tepidimicrobium (r = 0.183, *P* = 0.0165) were correlated with tumor size (Fig. 5i). Alistipes abundance was significantly associated with vessel invasion status (χ^2^ = 6.523, *P* = 0.0383), and Lysobacter abundance was related to HBV DNA status (χ^2^ = 9.226, *P* = 0.00240) (Fig. 5j). These findings indicated that intratumoral microbes could contribute in various ways to the prognosis of HCC who underwent surgical resection (Table S7).

## DISCUSSION

In a recent study by Nejman et al., the author detected intratumoral bacteria in seven cancer types, namely, breast, lung, ovary, pancreas, melanoma, bone, and brain tumors (15). They found that bacterial RNA and LPS were present in both cancer and immune cells among all human solid tumors examined. By utilizing fluorescently labeled D-alanine, they detected live intracellular bacteria in human breast tumors. In another study, Fu et al. confirmed that intratumoral bacteria were culturable and that the colonization of intratumoral bacteria inhibited lung metastasis in a breast tumor mouse model (27). Regarding HCC, Xue et al. revealed the difference in the detection of intratumoral bacteria between HCC tissues and adjacent normal tissues from mouse models and explored the potential association of the abundance of intratumoral bacteria with differential expression of genes and abundance of metabolites (18, 19). These results demonstrated the functional significance of intratumoral bacteria. However, due to the low biomass of the intratumoral microbiome and the unique characteristics of the tumor microenvironment, which are anaerobic and anacid, studies to determine the exact role of the intratumoral microbiome in tumor progression are still limited.

In this study, we investigated the microbiome composition and diversity of 172 HCC tumors obtained from patients at the Mengchao Hepatobiliary Hospital of Fujian Medical University. To identify the tumor region and bacteria, we employed multiple techniques, including the 5R multiplexed bacterial 16S rRNA gene sequencing technique, which provides species-level resolution. We confirmed the presence of LPS and LTA in tumor-associated immune cells.

We explored the intratumoral microbiome in the LOS and SOS groups. These results underscored the role of the intratumoral microbiota in predicting the prognosis of HCC patients who underwent surgical resection. Notably, we identified distinct tumor microbiome signatures characterized by specific bacteria between the LOS and SOS groups. Currently, there are many risk factors for prognosis determination after surgical resection, including tumor size, residual lesions, vascular invasion, HBV/HCV replication, liver cirrhosis degree, and the presence and degree of portal hypertension (28–30). An advanced histological grade of HCC tumors larger than 5 cm and a high incidence of vascular invasion are considered key factors and usually predict poor outcomes. However, more biological predictors of poor prognosis should also be considered. In this study, we found that similar to tumor size and vascular invasion, the intratumoral microbiome was highly predictive of prognosis for HCC patients who underwent surgical resection. We identified specific bacteria that serve as predictive markers of survival. The abundance of Intestinimonas in the SOS group was notable, while Brachybacterium and Rothia were significantly enriched in the LOS group. These three taxa were identified as independent risk factors for the prognosis of HCC patients who underwent surgical resection. Intestinimonas, commonly found in the intestinal tract, produce short-chain fatty acids (SCFAs) and secondary bile acids (31–33). SCFAs are associated with maintaining intestinal integrity and influencing hepatic metabolism through the AMPK pathway (34, 35). However, Brachybacterium has been less frequently reported to cause disease in recent decades. *Brachybacterium* spp. have been sporadically isolated from human blood culture, stool, and endophthalmitis (36–38). On the other hand, Rothia, a Gram-negative bacterium, has been linked to distant metastasis in squamous cell carcinoma (39) and was found to be less abundant in the oral cancer group than in the control group (40). Future investigations are warranted to elucidate their roles in either enhancing or impairing the immune response against tumors.

To summarize, our study not only revealed bacteria within HCC tumors but also yielded the differences in microbiota between the LOS and SOS groups. These findings elucidated the potential of the intratumoral microbiome as a prognostic biomarker. However, the results reported herein should be considered in the light of several limitations. First, due to ethical considerations, acquiring complete normal liver tissues is often unattainable, and the unique signature of the intratumoral microbiome needs to be explored. Second, diverse microbiomes are present inside tumor tissues, and it remains unclear whether they are present in the tumor cells or only in the immune cells compartment. Most importantly, their exact role in tumor progression is still poorly known.

### Conclusions

In conclusion, our study confirms the presence of bacteria inside HCC tissues and highlights the potential of the intratumoral microbiome as an effective predictor of prognosis for HCC patients who underwent surgical resection.

## MATERIALS AND METHODS

### Sample collection

The cohorts consisted of 344 clinical tissue specimens from 172 individuals, including 172 HCC tissues and paired paracancerous tissues that were resected 2 cm away from the tumorous tissues. All samples were collected from patients who underwent hepatectomy at Mengchao Hepatobiliary Hospital of Fujian Medical University. Written informed consent was obtained from all patients. The study was approved by the Ethics Committee of Mengchao Hepatobiliary Hospital of Fujian Medical University (approval no. 2021_100_02).

### Hematoxylin and eosin staining

H&E staining was performed following a standardized protocol as follows. Tissue samples were first fixed with formalin. Next, the fixed tissues were embedded in paraffin wax and thinly sliced into sections using a microtome. Third, the paraffin was removed from the tissue sections using xylene, followed by rehydration through a series of graded alcohol solutions. The tissue sections were stained by immersion in hematoxylin solution, followed by eosin solution staining. Finally, the stained tissue sections were dehydrated in graded alcohol solutions, cleared in xylene, and mounted on slides using a mounting medium. The sections were segregated based on the microscopic assessment of the percentage of hepatic area occupied by invasive cancer.

### Fluorescence *in situ* hybridization

Slides were stained for bacteria using an automated BOND RXm slide stainer (Leica) with Bond polymer to refine the detection following the kit manufacturer’s instructions. Heat-induced epitope retrieval at pH 6 was performed through a 20 min heating step with epitope retrieval solution 1 (BOND). FISH was carried out using a Vysis IntelliFISH Universal FFPE Tissue Pretreatment and Wash Reagents Kit (Abbott Molecular, Inc., IL). FFPE tumor tissues (4 µm thick) were hybridized with the probe EUB338 (5'-GCTGCCTCCCGTAGGAGT-3'), which targets bacterial 16S rRNA (stained green) (41), and counterstained with DAPI to visualize nuclei (stained blue). The tissues were visualized using a Nikon Eclipse Ti microscope.

### Multiplex immunofluorescence staining

Staining was conducted manually using primary antibodies against the following markers: CD45 (anti-CD45, eBioscience #14-0459-82), CD68 (anti-CD68, Invitrogen #MA5-12407), LPS (anti-LPS, abcam #ab35654), and LTA (anti-LTA, Santa Cruz #sc57752). The Opal Polymer HRP Ms + Rb detection reagent (PerkinElmer, Boston, MA), which features four reactive fluorophores (Opal 570, Opal 480, Opal 620, and Opal 690), was utilized for primary antibody detection. DAPI was added for nuclear counterstaining following the manufacturer’s instructions. Staining was performed consecutively, with each marker detected before the application of the next antibody. Uniplex IF and negative controls were stained following the same protocols. After staining, the slides were imaged using a Vectra 3.0 spectral imaging system (PerkinElmer).

### DNA extraction and amplicon sequencing

DNA was extracted with the TGuide S96 Magnetic Soil/Stool DNA Kit (Tiangen Biotech [Beijing] Co., Ltd.) following the manufacturer’s instructions. The DNA concentration of the samples was measured with a Qubit dsDNA HS Assay Kit and a Qubit 4.0 Fluorometer (Invitrogen, Thermo Fisher Scientific, Oregon, USA). The 338F: 5'-ACTCCTACGGGAGGCAGCA-3' and 806R: 5'-GGACTACHVGGGTWTCTAAT-3' universal primer set was utilized to amplify the V3-V4 region of the 16S rRNA gene from the genomic DNA extracted from each sample. Both the forward and reverse 16S primers were tailed with sample-specific Illumina index sequences to enable high-throughput sequencing. PCR was conducted in a total reaction volume of 10 µL: 5–50 ng of DNA template, 0.3 µL of *Vn F (10 µM), 0.3 µL of *Vn R (10 µM), 5 µL of KOD FX Neo Buffer, 2 µL of dNTPs (2 mM each), and 0.2 µL of KOD FX Neo, with ddH2O up to 10 µL. Vn F and Vn R were selected based on the amplification region. After initial denaturation at 95°C for 5 min, the mixture was subjected to 25 cycles of denaturation at 95°C for 30 s, annealing at 50°C for 30 s, and extension at 72°C for 40 s, with a final step at 72°C for 7 min. The PCR amplicons were then purified with Agencourt AMPure XP Beads (Beckman Coulter, Indianapolis, IN) and quantified using the Qubit dsDNA HS Assay Kit and a Qubit 4.0 Fluorometer (Invitrogen, Thermo Fisher Scientific, Oregon, USA). Following individual quantification, amplicons were pooled in equal amounts. For library construction, an Illumina NovaSeq 6000 (Illumina, Santiago CA, USA) was used for sequencing.

### Microbiome analysis pipeline

Raw paired-end 16S rRNA reads (from the V4 region) were merged into consensus fragments by FLASH (42) and subsequently filtered for quality (targeted error rate < 0.5%) and length (minimum 200 bp) using Trimmomatic (43) and QIIME (44, 45). Spurious hits to the PhiX control genome were identified using BLASTN and removed. Reads that passed initial quality control were trimmed of primer sequences, evaluated for chimeras with UCLUST (*de novo* mode) (46), and screened for human-associated contaminants using Bowtie2 (47). Chloroplast and mitochondrial contaminants were detected and filtered using the RDP classifier (48) with a confidence threshold of 50%. High-quality 16S rRNA sequences were assigned to a high-resolution taxonomic lineage using Resphera Insight (49, 50) and SILVA Database v128 (51). Bacterial contaminant removal was performed using four paraffin-only samples (no tissue) and a reference to the literature. The resulting contaminant-free 16S rRNA profiles were subsampled to 2,000 sequences per sample for downstream comparative analysis.

### Statistical analysis

All analyses were conducted using R-4.1.0 software. The fluorescence intensity was quantified by using ImageJ (52). Comparisons of diversity and microbial composition between the two groups were performed by the Wilcoxon rank sum test. A comparison of predicted biological functions between the two groups was performed by Student’s t-test. Linear discriminant analysis (LDA) effect size (LEfSe) was performed to determine the genomic features most likely to explain differences between biological classes (53). Multivariate Cox regression analysis was performed to identify independent risk factors for overall survival (54). Fisher’s exact test was performed to evaluate the associations between two categorical variables (55). Kaplan‒Meier curves were estimated for survival distributions. The log-rank test was used to test the difference in survival distributions between subgroups (56–58). *P* values less than 0.05 were considered to indicate statistical significance.

## Data Availability

The completed STORMS checklist is available at https://zenodo.org/records/13907014. The raw sequence data reported in this paper have been deposited in the GenBank Sequence Read Archive. Reads are available under accession number PRJNA1125920.

## SUPPLEMENTAL MATERIAL

Fig. S1 msystems.00765-24-s0001.tif Consecutive slices from HCC samples were stained with H&E or with FISH probes against bacterial RNA at 20×.

Fig. S2 msystems.00765-24-s0002.tif Representative images of multiplex immunofluorescence (multiplex IF, 20×) staining with an Opal kit.

Legends msystems.00765-24-s0003.docx Supplemental material legends.

Supplemental Tables msystems.00765-24-s0004.xlsx Tables S1 to S7.

ASM does not own the copyrights to Supplemental Material that may be linked to, or accessed through, an article. The authors have granted ASM a non-exclusive, world-wide license to publish the Supplemental Material files. Please contact the corresponding author directly for reuse.
