# Supplementary figures and images for: Association of intratumoral microbiome diversity with hepatocellular carcinoma prognosis
Source: mSystems. 2024 Dec 11;10(1):e00765-24. doi: 10.1128/msystems.00765-24 (PMC11748501; doi:10.1128/msystems.00765-24)

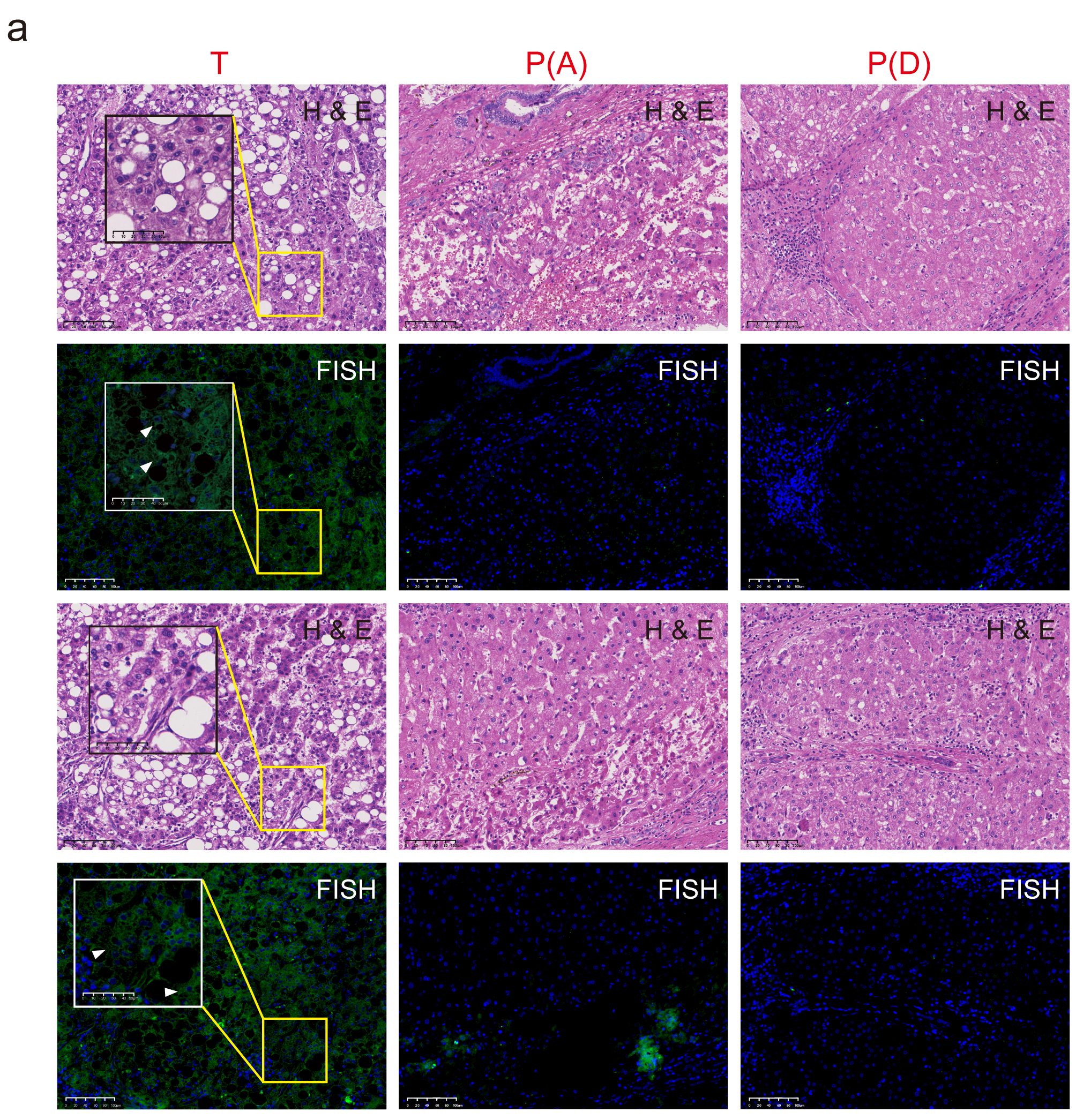

Supplement: Fig. S1 — Consecutive slices from HCC samples were stained with H&E or with FISH probes against bacterial RNA at 20×. [file msystems.00765-24-s0001.tif]

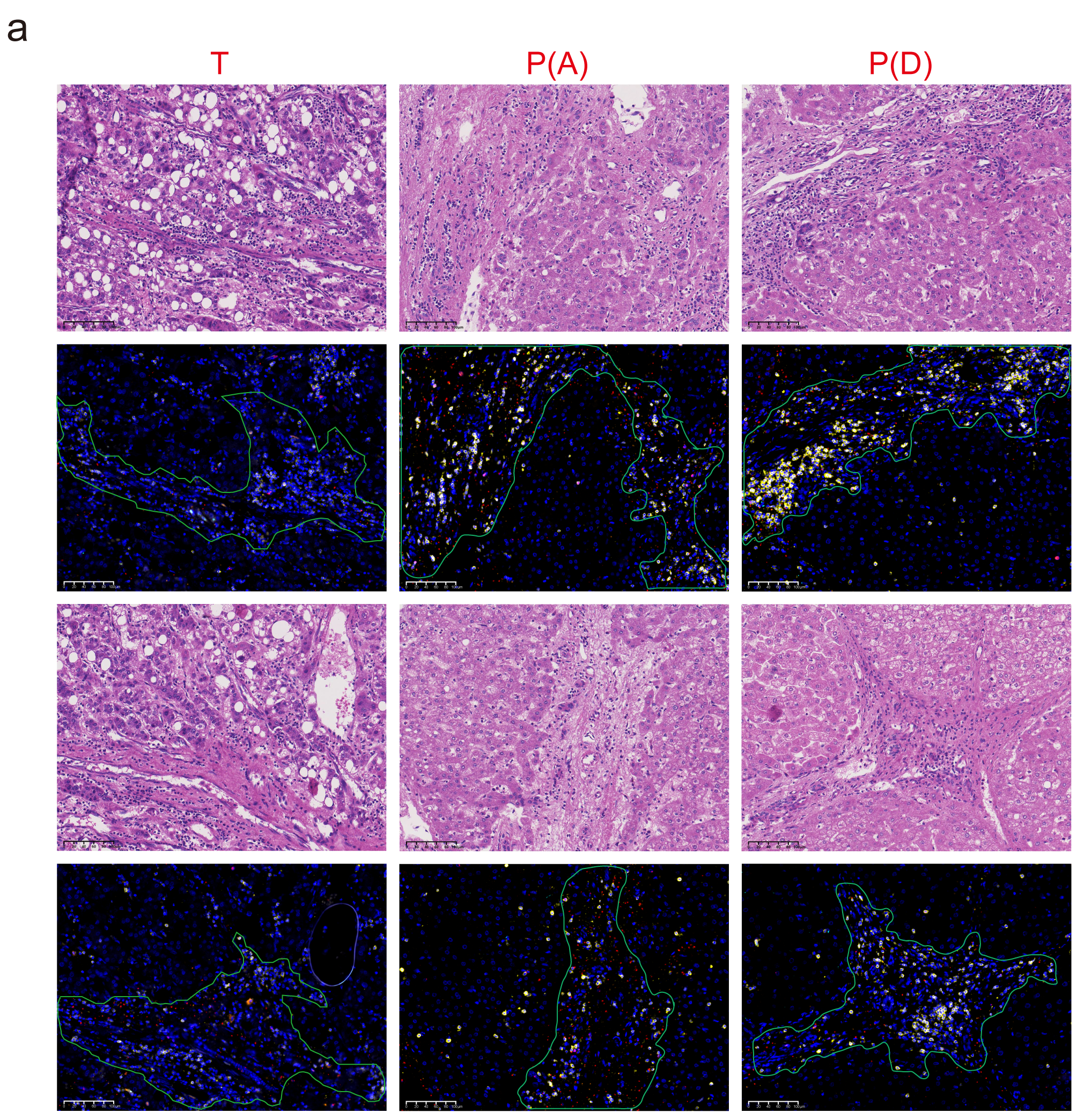

Supplement: Fig. S2 — Representative images of multiplex immunofluorescence (multiplex IF, 20×) staining with an Opal kit. [file msystems.00765-24-s0002.tif]
