# Supplementary material for: Association of intratumoral microbiome diversity with hepatocellular carcinoma prognosis
Source: mSystems. 2024 Dec 11;10(1):e00765-24. doi: 10.1128/msystems.00765-24 (PMC11748501; doi:10.1128/msystems.00765-24)
Supplement: Legends — Supplemental material legends. [file msystems.00765-24-s0003.docx]

Supporting information to Association of intratumoral microbiome diversity with hepatocellular carcinoma prognosis

SUPPLEMENTARY INFORMATION

The online version contains supplementary figures and tables available.

| Files | Title |
| --- | --- |
| Figure S1 | Consecutive slices from HCC samples were stained with H&E or with FISH probes against bacterial RNA at 20×. Bacterial 16S rRNA were stained in green, and cellular nuclei were stained in blue. The yellow box indicates the region enlarged in the low right. The white arrows indicated the 16S rRNA signal in the slices. T, Tumor tissue; P(A), nonneoplastic tissue (adjacent); P(D), nonneoplastic tissue (distant). Scale bars, 50 μm. |
| Figure S2 | Representative images of multiplex immunofluorescence (multiplex IF, 20**×**) staining with an Opal kit. The bacteria were stained with antibodies against LPS and LTA, while CD45 and CD68 were used to identify immune cells. The area circled by the green curve indicated the immune cell rich region (IRR). Scale bars, 50 μm. |
| Table S1A | The result of 16S rRNA gene sequencing (GC_Q statistics). |
| Table S1B | The result of 16S rRNA gene sequencing (OTU statistics). |
| Table S1C | The result of 16S rRNA gene sequencing (taxa statistics). |
| Table S2 | The intensity of 16s rRNA (FISH). |
| Table S3A | The intensity of LPS (mIF). |
| Table S3B | The intensity of LTA (mIF). |
| Table S4 | PICRUSt2 combined with the KEGG database to predict the function of bacterial microbiota in LOS and SOS, showing the results of the level 3 KEGG pathway analysis. |
| Table S5 | LEfSe analysis of the microbiota of LOS and SOS. |
| Table S6 | The association of three taxa with the mortality of the HCC subjects. |
| Table S7 | The association of the key taxa with the clinicopathological features of the HCC subjects. |
